# Retinal Protection from LED-Backlit Screen Lights by Short Wavelength Absorption Filters

**DOI:** 10.3390/cells10113248

**Published:** 2021-11-19

**Authors:** Celia Sanchez-Ramos, Cristina Bonnin-Arias, Vanesa Blázquez-Sánchez, Victoria Aguirre-Vilacoro, Teresa Cobo, Olivia García-Suarez, María Jesús Perez-Carrasco, Cristina Alvarez-Peregrina, José A. Vega

**Affiliations:** 1 Grupo de Neuro-Computación y Neuro-Robótica, Facultad de Óptica y Optometría, Universidad Complutense de Madrid, 28037 Madrid, Spain; celiasr@opt.ucm.es (C.S.-R.); cbonnina@ucm.es (C.B.-A.); vblazquez@ucm.es (V.B.-S.); victoriaguirre@telefonica.net (V.A.-V.); 2 Departamento de Cirugía y Especialidades Médico-Quirúrgicas, Universidad de Oviedo, 33006 Oviedo, Spain; teresacobo@uniovi.es; 3 Grupo SINPOS, Departamento de Morfología y Biología Celular, Universidad de Oviedo, 33006 Oviedo, Spain; garciaolivia@uniovi.es (O.G.-S.); javega@uniovi.es (J.A.V.); 4 Departamento de Óptica II: Optometría y Visión, Facultad de Óptica y Optometría, Universidad Complutense de Madrid, 28037 Madrid, Spain; mjpc@opt.ucm.es; 5 Faculty of Biomedical and Health Sciences, Universidad Europea de Madrid, 28670 Madrid, Spain; 6 Facultad de Ciencias de la Salud, Universidad Autónoma de Chile, Santiago 7500912, Chile

**Keywords:** retinal light injury, LED screen, optical filter, retinal protection

## Abstract

(1) Background: Ocular exposure to intense light or long-time exposure to low-intensity short-wavelength lights may cause eye injury. Excessive levels of blue light induce photochemical damage to the retinal pigment and degeneration of photoreceptors of the outer segments. Currently, people spend a lot of time watching LED screens that emit high proportions of blue light. This study aims to assess the effects of light emitted by LED tablet screens on pigmented rat retinas with and without optical filters. (2) Methods: Commercially available tablets were used for exposure experiments on three groups of rats. One was exposed to tablet screens, the other was exposed to the tablet screens with a selective filter and the other was a control group. Structure, gene expression (including life/death, extracellular matrix degradation, growth factors, and oxidative stress related genes), and immunohistochemistry in the retina were compared among groups. (3) Results: There was a reduction of the thickness of the external nuclear layer and changes in the genes involved in cell survival and death, extracellular matrix turnover, growth factors, inflammation, and oxidative stress, leading decrease in cell density and retinal damage in the first group. Modulation of gene changes was observed when the LED light of screens was modified with an optical filter. (4) Conclusions: The use of short-wavelength selective filters on the screens contribute to reduce LED light-induced damage in the rat retina.

## 1. Introduction

As early as 1966 it was suggested that exposure to low-intensity short-wavelength light for a long time can induce retinal damage [1], with the action spectrum (400–440 nm) of blue light the most dangerous [2,3], able to trigger or exacerbate macular and retinal damage [4,5]. Both human and animal studies suggest that excessive levels of blue light induces immediate photochemical damage to the retinal pigment epithelial cells (RPE), photoreceptors, and ganglion cells [2,3,6,7,8,9,10]. Thus, the phototoxicity of blue light may contribute to the progression and severity of age-related macular degeneration (AMD) and vision loss.

Nowadays, light-emitting diodes (LEDs) are gradually becoming the majority of the domestic light sources, replacing conventional ones [2,11,12]. The most commonly used method to produce white LED lights is the use of blue diodes covered with a phosphor compound; thus, one of the biggest risks of LEDs is its emission spectrum, since it contains a large blue light component [2,12,13]. Additionally, there is time-related degradation of phosphorus which leads to a progressively increasing release of short wavelengths (blue light) [9]. Since LEDs do not directly emit ultraviolet (UV) and infrared (IR) wavelengths, the blue light risk is the main ones to focus on when considering LEDs and LED systems [12].

Recent studies assessing LED effects on the retina using ambient LED lighting have demonstrated LED phototoxicity [2,3,9,11,12,14,15]. To our knowledge, no data are available regarding backlit LED screens. People spend increasing amounts of time viewing backlit LED screens, such as personal computers, tablets, smartphones, etc., at short distances. Thus, this raised a range of public concerns about their potential risks as retinal hazards [16,17,18]. In 2017, Lin et al. investigated the effects of periodic exposure to the blue LED in rats showing marked retinal damage [19] characterized by an accumulation of macrophages (identified because express ionized calcium binding adapter molecule-1), drusen-like materials around the outer segments of the photoreceptors, and finally degeneration of the photoreceptors [7]. Additionally, LED induce strong damage in rat retinal pigment epithelium (RPE) characterized by the breakdown of the blood–retinal barrier and the induction of necrotic cell death [20]. Furthermore, Balb/c mice exposed to LED light show a reduction of outer nuclear layer (ONL), increase in TUNEL-positive apoptotic nuclei, changes in several differentially expressed genes, and downregulation of ubiquitin and autophagy function [21]. At the basis of those changes is the oxidative stress induced by LED [22] which affects mitochondria and triggers mitochondrial death signaling pathways [17,23], as well as accumulation of damaged lysosomes and subsequent lysosomal cell death [24].

On the other hand, the deleterious effects of LED lights on the retina can be prevented partially by blue-light-blocking films [25], or more effectively by lenses with a brown or gray tint [26].

Previous studies from our research group have demonstrated that exposure to blue light reduces the number of retinal cells, upregulates genes related to cell death, downregulates genes involved in cell survival, increases the activity of genes involved in extracellular matrix degradation, and alters the expression of growth factor that participates in cell maintenance and survival. These effects can be prevented or reversed by filtering blue light [27,28]. Therefore, this study aims to evaluate the effects of light emitted by back-lit LED tablet screens on the retina of pigmented rats and determine whether or not they can be modified by partially filtering out the emitted short-wavelengths of the visible spectrum. Based on the multiple pathways that can participate in LED-induced retinal damage, and our previous experience and studies, the following genes were analyzed: genes involved in cell survival and death (Bcl-2; Bcl-XL; Bax; Bak; Bcl-XS, Caspases-3 and 9), genes related to the extracellular matrix (ECM) turnover (MMPs-2 and 9, ADAMTS-12 and 14, TIMPs-1 and 2), genes related to growth factors (BDNF-Trk-B system, VEGF-VEGFr-2 system), and inflammation (TNF-α, SODs-1 and 2). Furthermore, immunohistochemistry was used to study determined the protein product of the genes showing greater variation after exposure to LED. The results could support the effects of short-wavelengths emitted by back-lit LED screens on gene regulation and assess the efficacy of the filter in removing excessive blue light radiation, thus potentially providing a retinal photoprotective effect.

## 2. Materials and Methods

### 2.1. Animals and Rearing Conditions

Male Lister-Hooded rats obtained from Harlan Laboratories Models, S.L. (Barcelona, Spain), were housed at the bioterium of the Medical School of the Universidad Complutense de Madrid (UCM, Madrid, Spain). The animals were kept in a dark environment for 14 days to remove the effect of light exposure from their previous breeding location, with access to food and water ad libitum. The use of rats complied with the Statement for the Use of Animals in Ophthalmic and Vision Research (ARVO 2013). Animals were treated humanely and with regard to the alleviation of suffering. This study has the approval of the Animal Experimentation Committee of the UCM and the Department of Health of the Comunidad de Madrid, Spain (Reference PROEX 310/15).

Rats were divided into three groups, with 12 animals per group. Group 1 was exposed to the light emitted by the LED-backlit tablet screens; group 2 was exposed to the light emitted by the LED-backlit tablet screens with a selective short-wavelength absorption filter adhered to the screen; the control group was unexposed to LED-backlit tablet screens. Animals were housed in clear Makrolon Polycarbonate cages, 58 × 38 × 18 cm in size, with two animals per cage.

### 2.2. Light Source and Exposure

Light emitted by blank LED-backlit tablet screens, with size 231 × 147.2 × 8.7 mm (Cristal IPS multitouch 8.9”, 1920 × 1200 px Full HD), set at full brightness was used. The selective short-wavelength absorption filter used for group 2 exposure conditions was Reticare^®^ Intensive (Tecnología Sostenible y Responsable S.L., Madrid, Spain). Before starting the study, the filter was adhered to each tablet screen, following the manufacturer’s instructions.

The photo exposure process was designed aiming at simulating the conditions of use of touch screens by children. The screens were placed surrounding the cages, at a distance between 4.72 and 5.90 inches of the animal’s eyes, leaving the upper and lower areas without screens. As for the devices used for photo exposure, tablets were used instead of smartphones for two reasons: firstly, because the time of use of tablets, in general, is longer than smartphones; and secondly, because the tablets emit with less intensity (roughly half) than mobile phones.

**Figure 1:**
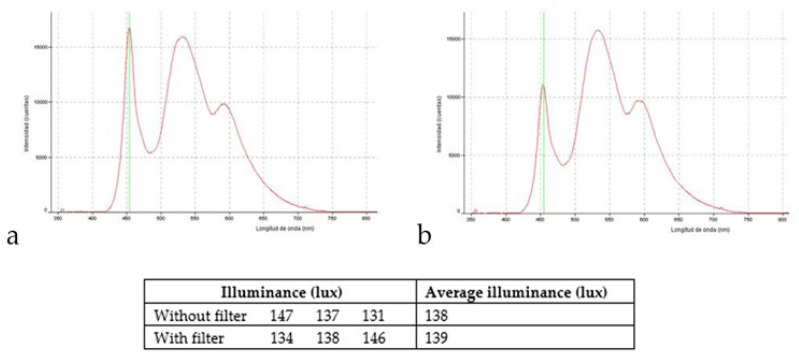
Characteristic of the light LED screen emission and the illuminance (lux) measured inside the study cages; (**a**) without optical filter; (**b**) with the optical filter.

Tablet screens were set at 10 cm from each of the four cage walls. For groups 1 and 2, each cage contained six screens (without or with filter, respectively), one screen in each of the two short walls, and two screens in each of the two long walls. No screens were attached to cage ceilings. Figure 1 shows the characteristic of the light LED screen emission and the illuminance (lux) measured inside the study cages, as well as the transmittance curve of selective short-wavelength absorption filter (Reticare^®^ Intensive). LED-backlit screen tablet light emission (with and without filter) was measured with an Ocean Optics USB2000 + Spectrometer.

Animals of groups 1 and 2 were exposed to 8-hr dark/16-hr LED-backlit screen tablet light cycles for three months. All animals, including a control group, were sacrificed at the end of the exposure period.

### 2.3. Tissue Collection, RNA Extraction, and qPCR

The animals were sacrificed with an overdose of pentobarbital sodium (200 mg/kg, ip) and eyes were removed. The left eyes of each animal were frozen at −80 °C and maintained until quantitative polymerase chain reaction (qPCR) analysis. Right eyes were fixed in 4% formaldehyde for 24 h, then washed in tap water, and routinely processed for paraffin inclusion, and used for structural and immunohistochemical studies.

Total RNA was extracted from the whole retina using a commercial kit (Trizol Reagent, Invitrogen, Carlsbad, CA, USA), following the manufacturer’s instructions. After precipitation and cold ethanol washing, RNA was dried and dissolved in an appropriate volume of Tris–EDTA buffer (10 mM Tris–HCl pH 8.0 and 1mM EDTA-Na2). Each sample was treated with 1U of DNase I for 1-h at 37 °C to digest genomic DNA. RNA was precipitated, washed, and dissolved again in the same buffer. RNA solution was quantified at 260 nm (Biomate 3, Thermo Electron Corporation, Waltham, MA, USA) and its purity was assessed by the ratio of 260/280 nm readings. We used the High Capacity cDNA Archive kit (Applied Biosystems, Foster City, CA, USA), random hexamers, and 10 µg of total RNA to make cDNA following the manufacturer’s instructions. Then, 1 µg of cDNA was used to detect the expression of the genes analyzed in this qPCR study.

Quantitative PCR was performed using 1 µg of the cDNA, and the primers used to detect the investigated genes, and the β-actin were designed based upon the published mRNA sequences for Rattus norvegicus (Table 1; GenBank accession numbers are included).

Homemade TaqMan probes were labeled at the 50 with 60 FAM fluorochromes for all investigated sequences, and VIC fluorochrome for β-actin, while the 3′ ends were labeled with the Minor Groove Binder (MGB) quencher. The PCR reactions were performed using the TaqMan Universal PCR Master Mix (Applied Biosystems, Foster City, CA, USA) using 5 pmol of each primer and 9 pmol of both target and β-actin probe. The assays were performed in triplicate in independent experiments using a 7500 PCR real-time system (Applied Biosystems), and quantification was calculated using the 2-∆∆Ct algorithm against β-actin and expressed as the n-fold difference compared to an arbitrary calibrator, chosen as a higher value than ∆∆Ct.

Average values obtained in control group animals were compared to those exposed to LED-backlit tablet screen light, without and with filter (group 1 and 2, respectively), and the results were expressed as fold difference compared to control (relative expression).

### 2.4. Structural and Immunohistochemical Studies

The structure of the retinal in all animal groups evaluated by staining representative formol fixed, paraffin embedded sections (10 per eye and animal) with hematoxylin & eosin. The sections were then scanned by an SCN400F scanner (Leica Biosystems™, Newcastle, UK), and the scans were computerized using SlidePath Gateway LAN software (Leica, Leica Biosystems™). Then, the whole thickness of the retina was measured, evaluating in 10 points the distance from the inner to the other surface of the retina.

Deparaffinized and rehydrated sections were processed for detection SYN using the EnVision antibody complex detection kit (DakoCytomation, Copenhagen, Denmark), following supplier’s instructions. Briefly, the endogenous peroxidase activity and non-specific binding were blocked, and the sections were then incubated overnight at 4 °C with the primary antibodies included in the Table 2. Subsequently, sections were rinsed incubated with Dako EnVision System labeled polymer-HR anti-mouse IgG (DakoCytomation) for 30 min at room temperature. Finally, sections were washed, and immunoreaction visualized using 3-3′-diaminobenzidine as chromogen. To ascertain structural details some sections were counterstained with Mayer’s hematoxylin, dehydrated, and mounted with Entellan^®^ (Merk, Dramstadt, Germany).

The variations in the intensity of immunostaining developed for each investigated antibody was evaluated in five sections per animal separated by 100 µm. Each section was scanned with an SCN400F scanner (Leica Biosystems™) and annotated using SlidePath Gateway LAN software (Leica Biosystems™). A 1 mm^2^ grid was then applied randomly onto 2 × 500 µm enlarged images in 4 non-overlapping fields (4 mm^2^ per section; 20 mm^2^ per subject), and the free nerve endings and sensory corpuscles within the grid were counted by two independent observers. The results are expressed as absolute numbers for the densities of the sensory corpuscles and free nerve endings per cm^2^. The intensity of immunostaining developed with the different retinal layers was evaluated in arbitrary units of grey levels ranging from 1 (black) to 256 (white) using an image analysis system (MIP System, Servicio de Análisis de Imágenes, Universidad de Oviedo, Spain). The intensities were therefore divided into four groups (64 units of grey level each), referred in the text and tables as strong (1–64, ++++), high (65–128, +++), intermediate (129–192), and low (193 to level of the background in control sections, +). Intensities of grey higher than those of background of the corresponding control sections were considered unreactive. Although we attempted to process all samples identically, there may have been variations in the intensity of the final immunoreaction due to technical aspects, i.e., differences in penetration of the fixative. On the other hand, for the immunohistochemical study, the retinal segment and orientation of the sections were not taken into account, and therefore the thickness of the retina may appear in the images with greater thickness in the experimental animals than in the controls.

### 2.5. Statistical Analysis

Significant differences among the three groups were assessed with the Kruskal–Wallis H test, and *p*-values < 0.05 were considered statistically significant (marked in the figures as * *p* < 0.05, ** *p* < 0.01).

## 3. Results

The study was carried out with 36 male Lister-Hooded rats obtained from Harlan Laboratories Models, S.L. (Barcelona, Spain), housed at the bioterium of the Medical School of the Universidad Complutense de Madrid (UCM, Madrid, Spain), with food and water ad libitum, kept in a dark environment for 14 days to remove the effect of light exposure from their previous breeding location. Rats were divided into 3 groups, with 12 animals per group. Group 1 was exposed to the light emitted by the LED-backlit tablet screens; Group 2 was exposed to the light emitted by the LED-backlit tablet screens with a selective short-wavelength absorption filter adhered to the screen; the control group was unexposed to LED-backlit tablet screens.

### 3.1. Structural Study

**Figure 2:**
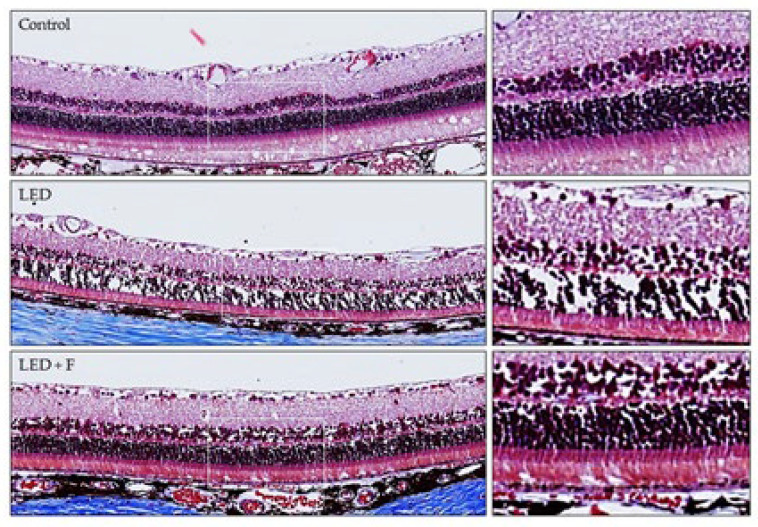
Retinal structure from control rats, rats exposed to LED Screen (LED-S), and rats exposed to LED screen with a protective filter (LED-S-P).

The retinal structure was studied in the three groups of animals. In those exposed to LED-backlit tablet screen light, there was a significant decrease in the whole retinal thickness (reduction of 23.82 ± 6.21%), apparently due to a reduction in the number of cells in both the outer and inner nuclear layers. The whole thickness of the retina was measured by evaluating in 10 points the distance from the inner to the other surface of the retina. These structural changes were almost reversed by the selective short-wavelength absorption filter (Figure 2).

### 3.2. Gene Expression Study

#### 3.2.1. Life/Death Cell-Related Genes

**Figure 3:**
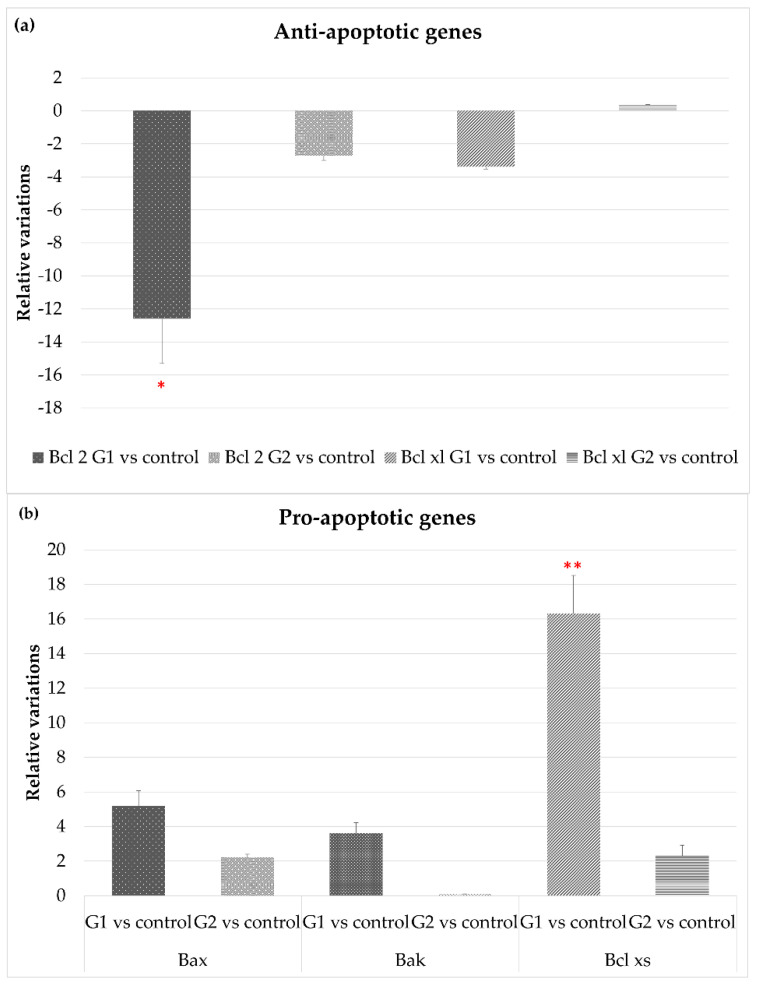

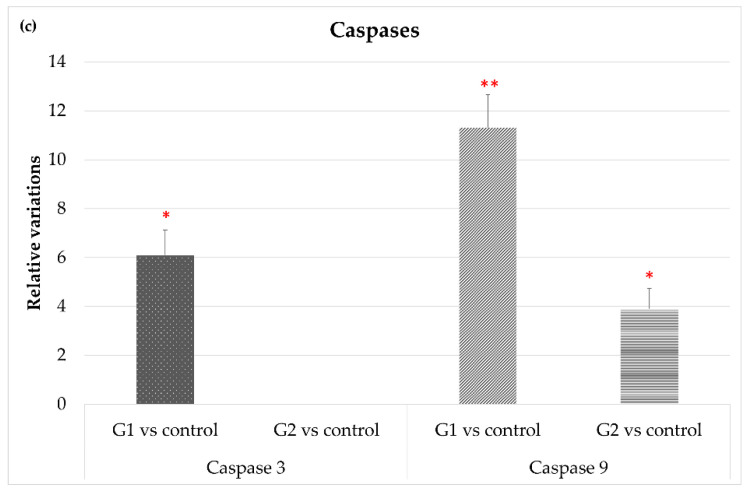
Gene expression comparisons between group 1 vs. control and group 2 vs. control. (**a**) Anti-apoptotic gene expression, (**b**) pro-apoptotic gene expression, and (**c**) gene expression of apoptotic-related enzymes. * *p* < 0.05; ** *p* < 0.01.

Different genes related to cell survival and cell death were analyzed. The eyes of the animals exposed to LED-backlit tablet screen light without filter (group 1) showed a down-regulation of the genes encoding anti-apoptotic proteins, especially Bcl-2 (−13.2-fold), and to a lesser extent Bcl-XL (−3.5-fold), as Figure 3a shows. Selective short-wavelength absorption almost eliminated these responses. Regarding genes directly or indirectly involved in cell death, exposition to the LED-backlit tablet screen light without filter resulted in a moderate up-regulation of Bax and Bak (5- and 3.3-fold, respectively) and a marked up-regulation of Bcl-XS (16.3 folds), as Figure 3b shows. Similarly, in this group (group 1), there was an up-regulation of both caspase-3 (6-fold) and caspase-9 (11.8-fold) genes. This scenario changes after the use of the selective short-wavelength absorption filter. In fact, in this experimental situation, Bak and caspase-3 were undetectable, and the expression of Bax, Bcl-XL, and caspase-9 remained up-regulated for the controls, but the expression was significantly reduced for the group of animals exposed to LED-backlit screens, as Figure 3c shows.

#### 3.2.2. Extracellular Matrix Degradation: MMP/TIMPs System and ADAMTS Genes

**Figure 4:**
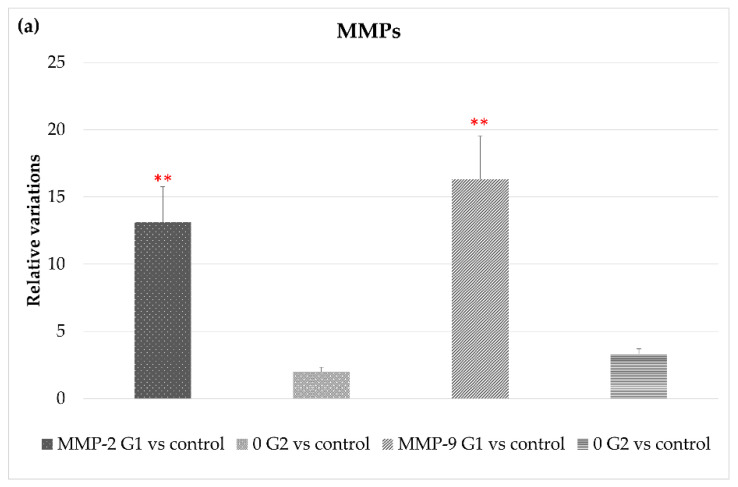

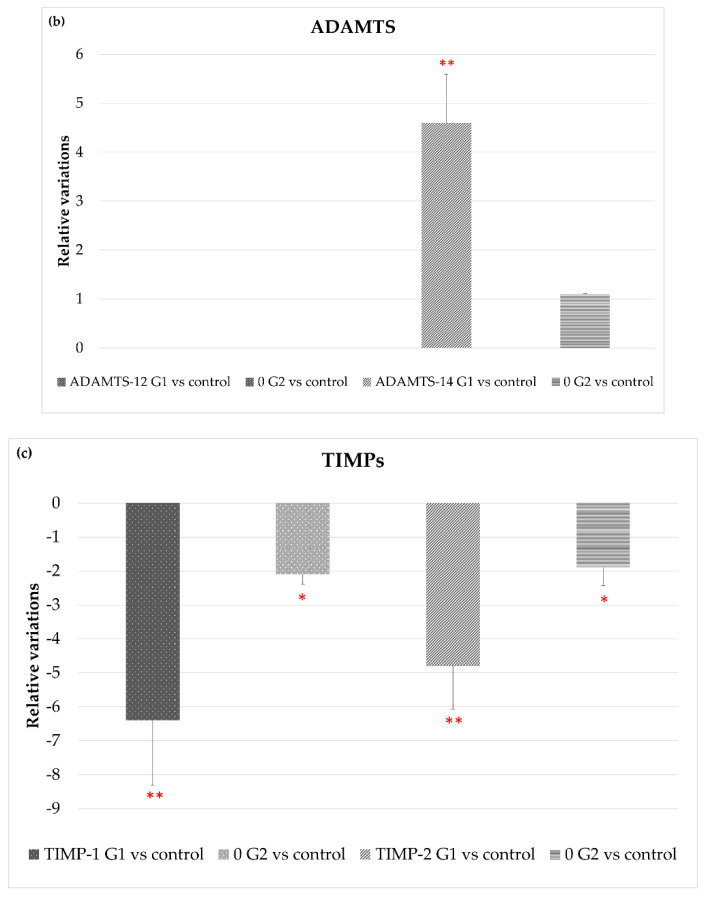
Variations in gene expression in both, experimental vs. control groups of (**a**) MMP2 and MMP9; (**b**) ADAMTS-12 and ADMSTS-14; and (**c**) TIMP1 and TIMP-2, * *p* < 0.05; ** *p* < 0.01.

Figure 4 shows that exposure to LED-backlit tablet screen light without filter (group 1) entails an increase in MMP-2 and MMP-9 mRNA expression, suggesting an increase in the extracellular matrix (ECM) turnover mediated by these proteases. As for changes in TIMPs expression, exposure to light emitted by LED-backlit tablet screens caused a decrease of both TIMP-1 and TIMP-2. The use of the selective short-wavelength absorption filter almost reversed the decrease, although in levels below control group values, as Figure 4c shows. Regarding ADAMTS-12, no differences were found between the control group versus either group 1 or group 2. On the other hand, exposure to LED-backlit tablet screen light without filter (group 1) increased the levels of ADAMTS-14 expression, whereas exposure effects were reverted with the filter (group 2), as Figure 4b shows.

#### 3.2.3. BDNF/TrkB and VEGF/VEGFR-2 System, and TNF-α

**Figure 5:**
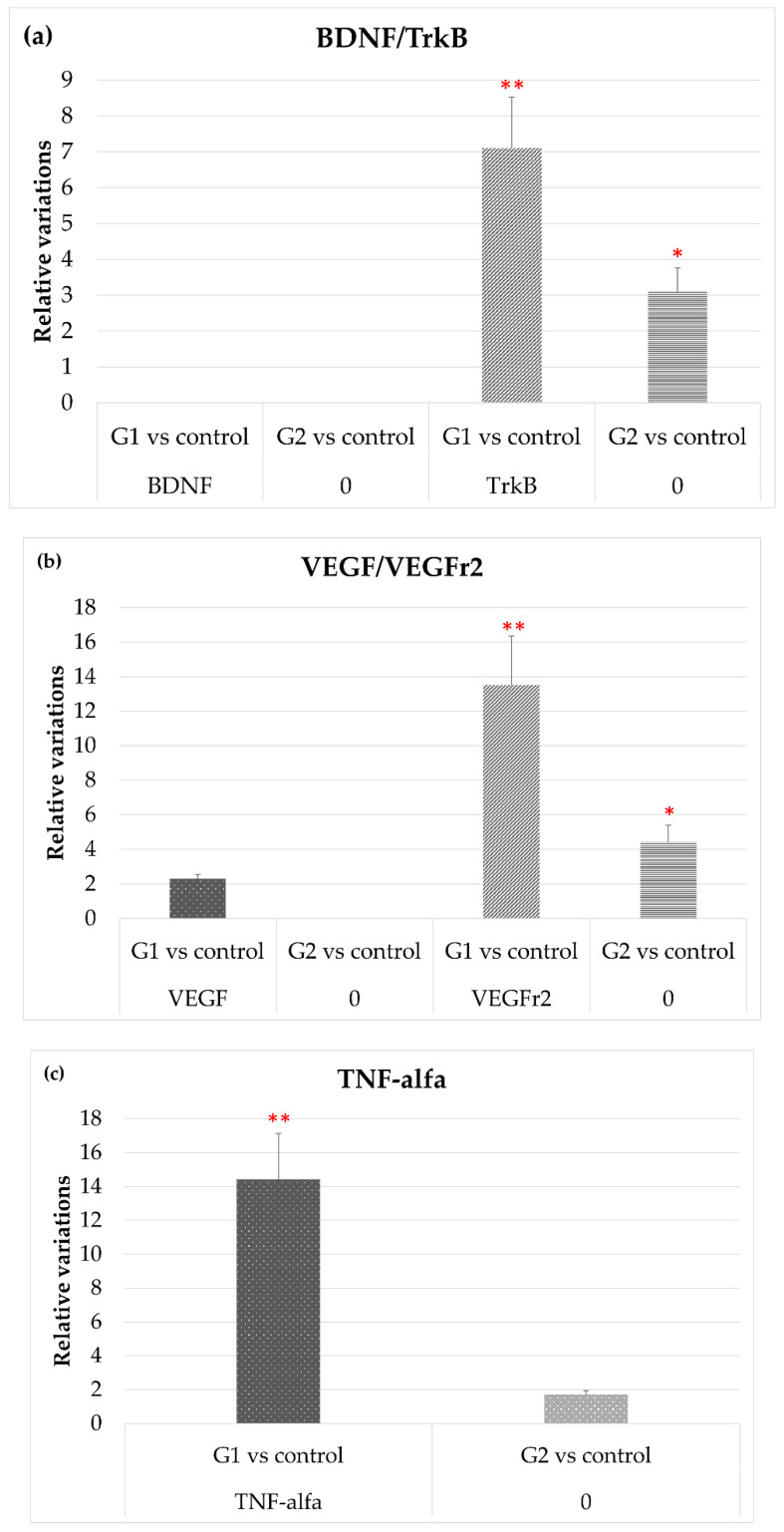
Variation of the expression in both experimental vs. control groups, of (**a**) TrKB; (**b**) VEHG and VEGFr2; and (**c**) and TNF-α. * *p* < 0.05; ** *p* < 0.01.

Figure 5a shows how exposure to LED-backlit tablet screen light did not modify expression levels of BDNF either with or without the selective short-wavelength absorption filter, while TrkB gene expression increased significantly. It is important to highlight that, with the filter, TrkB gene expression persisted at a high level, although below the level observed in group 1 (without filter).

Figure 5b shows that after exposure to LED-backlit tablet screen light (group 1), levels of VEGF mRNA increase moderately, while levels of its receptor increased by nearly 14-fold. These effects were highly attenuated using the selective short-wavelength absorption filter (group 2): VEGF levels were practically identical to controls, and VEGFr2 were lower, although above controls.

Finally, Figure 5c shows that TNF-α mRNA expression levels increased up to 14-fold in group 1 rats (without filter) but were like controls in group 2 (with filter).

#### 3.2.4. Oxidative Stress

**Figure 6:**
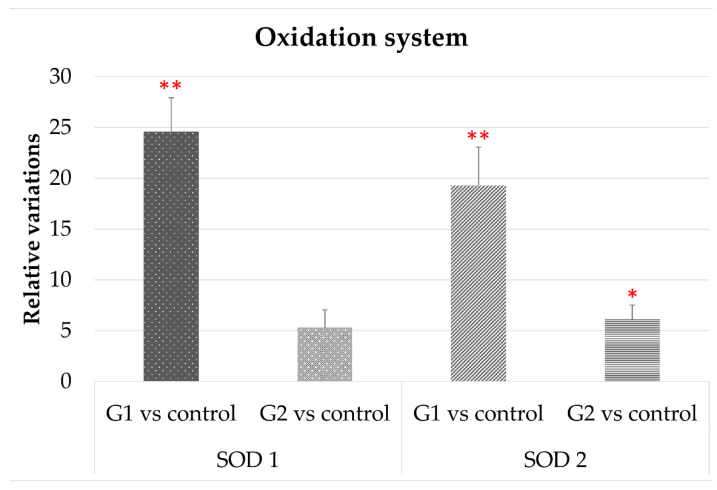
Variation in the expression of SOD1 and SOD2 in both experimental vs. control groups. * *p* < 0.05; ** *p* < 0.01.

Figure 6 shows how the expression of superoxide dismutase-1 and -2 were strongly up-regulated after exposure to LED-backlit tablet screen light (group 1). These effects are highly attenuated, but increased for the controls, using the selective short-wavelength absorption filter.

Table 3 summarizes the results of the variations in the expression of the genes analyzed, as a complement of the figures.

### 3.3. Immunohistochemistry

**Figure 7:**
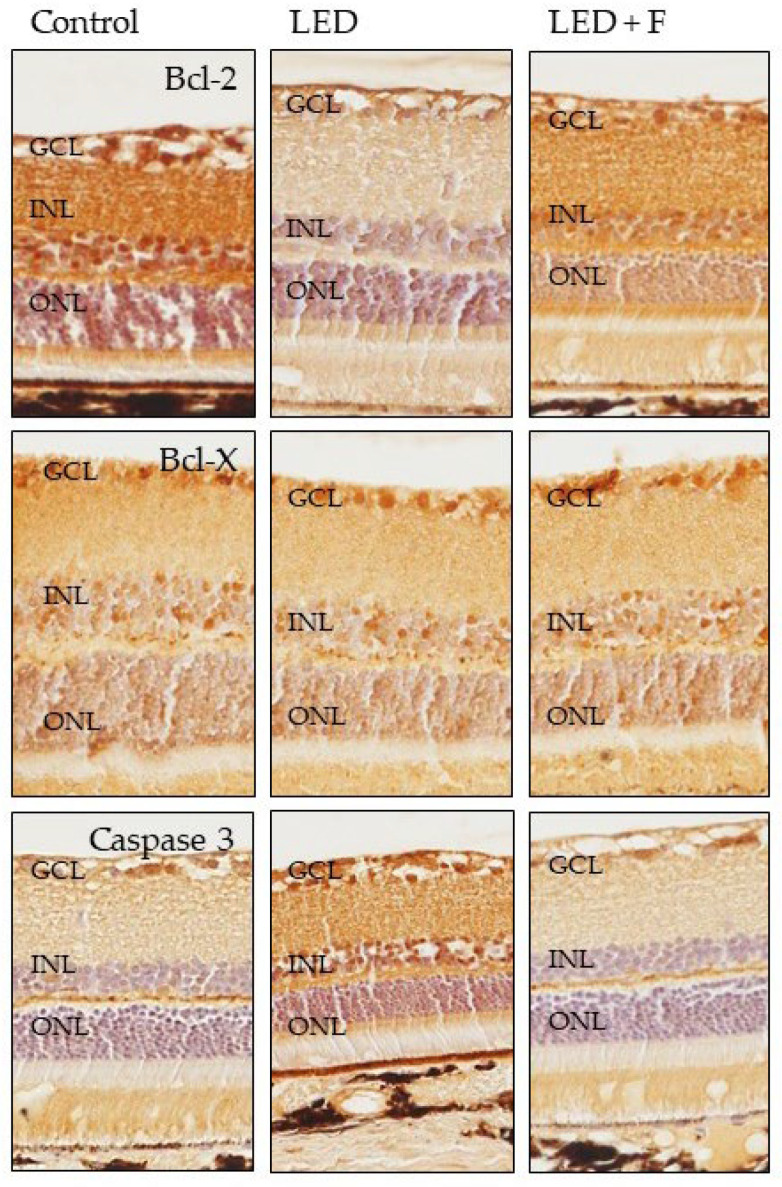
Immunohistochemical detection of Bcl-2 (**upper panel**), Bcl-X (**central panel**) and Caspase-3 (**lower panel**) in the three groups of rats investigated. LED: animals exposed to LED-backlit screen for 3 months; LED + F: animals exposed to filtered LED-backlit screen for 3 months. GCL: ganglionic cells layer; INL: inner nuclear layer; ONL: outer nuclear layer.

Using immunohistochemistry, we analyzed the detection in retinal sections of the protein products of the genes whose expression were most affected by the experimental conditions to which the two groups of animals. The increases or decreases in the intensity of immunostaining paralleled that observed in the gene expression. The immunolabelling for Bcl-2 was greatly reduced in animals exposed to LEDs and partially recovered when light was filtered (Figure 7), whereas no notable variations were observed for Bcl-X (Figure 7). The detection of caspase-3 showed an increase in immunostaining in the group subjected to LED light and distribution and intensity similar to the controls in animals subjected to filtered light (Figure 7).

**Figure 8:**
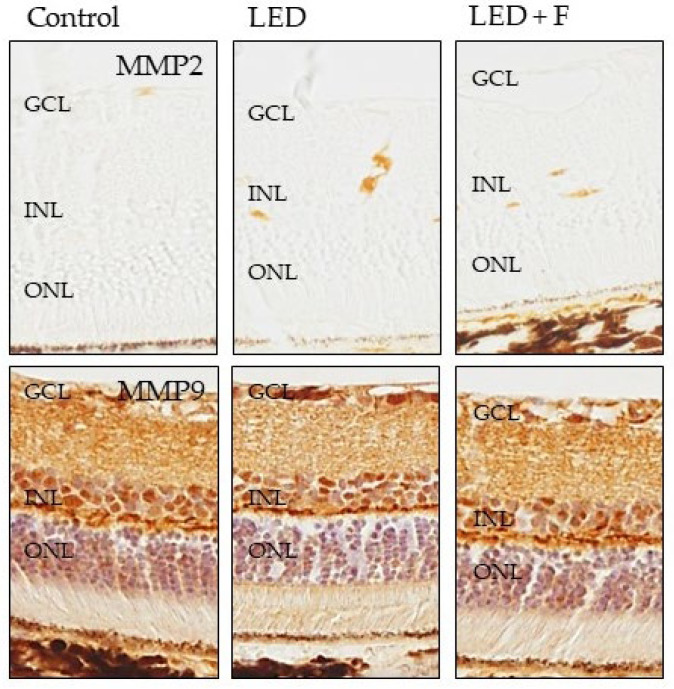
Immunohistochemical detection of MMP2 (**upper panel**), and MMP9 (**lower panel**) in the three groups of rats investigated. LED: animals exposed to LED-backlit screen for 3 months; LED + F: animals exposed to filtered LED-backlit screen for 3 months. GCL: ganglionic cells layer; INL: inner nuclear layer; ONL: outer nuclear layer.

Regarding the two metalloproteases investigated, MMP2 did not show positive immunoreaction in the control animals, while showing a slight immunopositivity in the animals subjected to LEDs that was reduced in the group of filtered LED light (Figure 8). On the other hand, the immunoreaction for MMP9 was very similar in the three groups (Figure 8).

**Figure 9:**
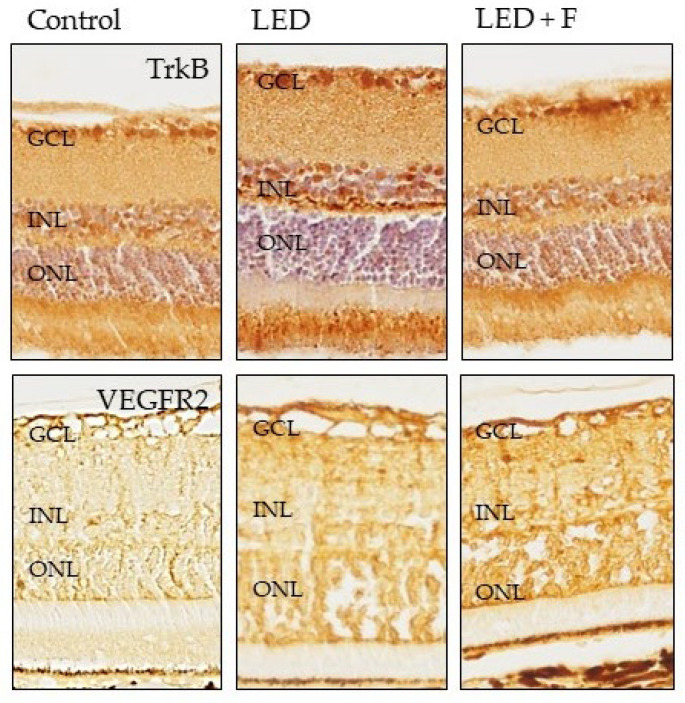
Immunohistochemical detection of TrkB (**upper panel**), and VEGFR2 (**lower panel**) in the three groups of rats investigated. LED: animals exposed to LED-backlit screen for 3 months; LED + F: animals exposed to filtered LED-backlit screen for 3 months. GCL: ganglionic cells layer; INL: inner nuclear layer; ONL: outer nuclear layer.

The immunoreactivity for TrkB, the high affinity receptor for the neurotrophin BDNF, was increased in animals exposed to LED, especially in the photoreceptors, but also in the inner nuclear layer and ganglionic cells layer, with levels similar to those of the animal controls when filtering LEDs (Figure 9).

**Figure 10:**
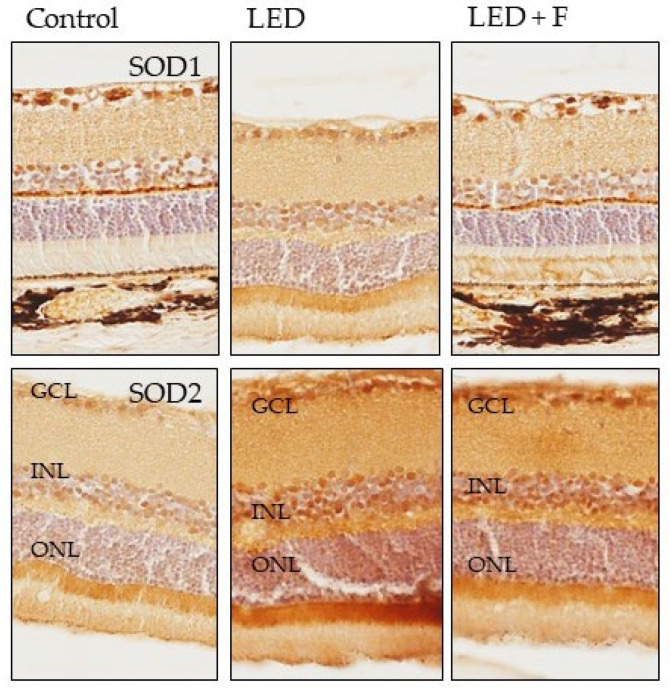
Immunohistochemical detection of SOD1 (**upper panel**), and SOD2 (**lower panel**) in the three groups of rats investigated. LED: animals exposed to LED-backlit screen for 3 months; LED + F: animals exposed to filtered LED-backlit screen for 3 months. GCL: ganglionic cells layer; INL: inner nuclear layer; ONL: outer nuclear layer.

The immunoreactivity for SOD1 increased in the photoreceptors layer of LED exposed rats and returned to similar levels of the control after LED filtering. However, SOD2 immunoreactivity increased in the photoreceptors, inner nuclear and ganglionic cell layers in LED-exposed animals, and the pattern of expression was not reverted by filtering (Figure 10).

The results of the quantitative study performed on immunohistochemical sections are in Table 4.

## 4. Discussion

The present study was designed to investigate the effects of the exposure to LED-backlit tablet screen light on the structure, gene expression, and protein localization of the retina of a rat model. Moreover, we have analyzed whether or not those effects could be reversed partial or using a selective short-wavelength absorption filter. The genes and proteins investigated were related to cell survival and cell death, as well as some proteases, and protease-blockers involved in the turnover of the ECM, growth factors previously known to be affected by light exposure, one involved in inflammation, and others related to oxidative stress. This approach is necessary because of the multiple factors involved in phototoxicity [15]. Because the results of the gene expression and immunohistochemistry were in parallel, they are discussed together.

In 2001, Dawson showed that blue LEDs (460 nm) and argon lasers (458 nm) induced retinal damage with corneal irradiances of 10 J/cm^2^ [29]. Ueda et al. also observed detrimental effects in the macula with blue LEDs (465 nm) [30]; besides, Shang et al. showed that the spectral range distribution of blue-white LEDs contains a significant fraction of short-wavelengths that induced irreversible retinal neuronal cell death in rats; they exposed Sprague-Dawley rats to white and blue LED light (750 lux) for 28 days, finding an increase in free radical production in the LED-exposed group [9]. Finally, Krigel et al. used albino Wistar and pigmented Long Evans rats exposed for 1–28 days to 500–6000 lux LEDs (cold white, blue, and green) [15]. The authors found that the blue component of the white-LED reduced photoreceptor layer thickness and induced retinal toxicity. In previous in vitro studies, Chamorro et al. exposed human retina cells to LED lighting in 12-h dark/12-h light cycles affected RPE cell growth and induced cellular stress, increasing levels of reactive oxygen species, DNA damage, and apoptotic cells [3]. Additionally, when a selective blue-light absorption filter was added, phototoxic damage in human RPE cells was reduced, providing a retinal photoprotector effect. However, few studies have investigated long-term and low luminance light-induced retinal phototoxicity and, to our knowledge, all have been carried out using different ambient LED lights. In contrast to previous studies, we investigated the induced retinopathy in a rat model due with long-term, low-intensity exposure to commercially available LED-backlit tablet light. Thus, the main objective of the current study was to assess the long-term effects on the retina of pigmented rats by light emitted by a backlit commercially-available digital device LED screen (tablet), with and without a short-wavelength selective absorption filter, in 16-h light/8-h dark cycles.

Although albino rats are commonly used for studies about retinal damage [9], in our study we have used pigmented rats. Compared to albino strains, the age of the animals at the onset of the light exposure, and not ocular pigmentation, is the most important factor in regulating the severity of light-induced retinal damage [31]. However, following the Shang et al. protocol, the conditions of the animals analyzed included a 14-day pre-study housing period in a dark environment to clear the light exposure effect prior to breeding conditions. Experimental animal retinas were baseline evaluated after this pre-study wash-out period. The experimental conditions of the present study differ substantially and significantly in various aspects from that of Shang et al. study [9]. Firstly, in the light source used: those authors used single-wavelength blue LEDs (460 ± 10 nm) and custom-made PC white LEDs at domestic lighting levels, while we have used commercially available LED-backlit tablet screens, to replicate real-life usage. Secondly, the exposure conditions: in our study, back-lit LED tablet screens exposed to rats with and without a selective short-wavelength absorption filter, aimed to show that, by the use of the filter, the transmission reduction of the most energetic LED light emitted band (blue light) resulted in reduced cell damage, as shown in studies conducted by Sparrow et al. and Nagai et al., who used yellow optic filtered intraocular lenses [32,33]. Thirdly, the mean luminance used by Shang et al. was 750 lux, the usual domestic luminance level, while in our study mean luminance was 140 lux, which corresponds to the light emitted by a commercially available LED-backlit tablet screen-blank screen set at full brightness. Finally, in the exposure time: whereas Shang et al. used light exposure times of 3, 9, and 28 days under 12-h dark/12-h light cycles, we extended the study to 3 months under more aggressive cycles: 8-h dark/16-h light.

To our knowledge, this is the first study regarding the effects of LED-backlit screens on the mammalian retina, and it is relevant because the time we spend watching LED-backlit screens of domestic devices (as personal computers, tablets, smartphones) at short distances has increased progressively in recent years, and this also includes infants and young people.

The retinal structure in the group of animals exposed to long periods of LED-backlit tablets light was altered, showing a significant decreases in the number of cells in the outer nuclear layer. No further significant changes were observed neither in the inner nuclear layer nor in the ganglion cell layer. These results agree with those obtained by Shang et al. in 2014 [9]. Lin et al. in 2017 reported marked retinal damage by regular exposure to a blue light-emitting diode in rats [19]. However, interestingly, these structural changes were prevented by filtering light specifically, as previously reported by different authors, including our research group.

The reduction in the number of retinal cells due to blue light exposure might be due to multiple factors, such as apoptosis of photoreceptors and RPE reported earlier [2,3,9,11,12,14,15]. Our results on the life/death-related genes further argue for the causes of cell loss after back-lit LED screens exposure. We observed that long periods of light exposure increase the expression of some genes related to cell death, such as Bax, Bak, and Bcl-XS as well as caspase 3 and caspase 9; conversely, the genes related to cell survival like Bcl-2 and Bcl-xl were down-regulated. Supporting our results, Lin et al. also observed increased expression of Bax and caspase-3, decreased expression of inhibited Bcl-2 and Bcl-xL, and inhibition of Bcl-2/Bax association in the RPE after regular exposure to blue light-emitting diode [19].

The exposition to LED-backlit screens strongly up-regulates MMP-2 and MMP-9, to a lesser extent ADAMTS-14, and was without effect on ADAMTS-12, and down-regulates both TIMP-1 and TIMP-2. These changes could have deleterious effects on the retina due to the extracellular matrix (ECM). Increased MMP expression could induce a faster ECM turnover to elude matrix deposit genesis [34]. Our experiments indicate upregulation in MMP-2 and MMP-9 expression, especially in rats exposed to the unfiltered LED tablet screen, whereas Sanchez-Ramos et al. in 2010 did not find any change in MMP-2 expression in other animal model trials [27]. Plantner et al. detected an increase in this MMP [35]. As for MMP-9 expression, our results are consistent with those obtained by Papp et al. and by Sanchez-Ramos [27,36]. In general, these results cannot support the hypothesis that the formation of deposits that give rise to retinal drusen is due to a decrease in pigmentary epithelium MMPs [37], as there are two possible interpretations. On one hand, long-term exposure to light causes an increase in the expression of some MMPs and ADAMTS-14 concurrently by a decrease of TIMP-1 and TIMP-2. These facts could affect the retina due to ECM damage. On the other, the increase in MMPs and ADMATS-14 expression could be related to increased ECM turnover to avoid the appearance of deposits that causes drusen [27].

As for the analysis of the results of BDNF and its receptor TrkB, exposure to light in our study conditions, whether with or without filter, had no effects on BDNF gene expression, which is consistent with findings by Wen et al., who reported that continuous exposure to white light had no significant effects on BDNF expression [38]. Asai et al. also obtained similar results by Western blot densitometry [39]. On the contrary, TrkB receptor expression levels were affected by LED-backlit screen tablet light in rat retinas, both with and without the filter. However, compared to filtered conditions, gene change was greater when exposure was without the filter. Given TrkB essentially has a survival and protector role, the increased levels of TrkB could be related to retinal protection against phototoxicity [40]. Interestingly, maximum TrkB increases were obtained in rats exposed to light in unfiltered conditions, with a higher proportion of short wavelengths, the most deleterious for the retina [41]. Therefore, an increase in TrkB expression, after LED-light exposure, could be considered as a neuroprotection response. On the other hand, photoreceptor integrity and survival could be irreversibly compromised by the short-term stress of the RPE. Exposure to intense visible light activates the VEGF signal and, thus, the breakdown of the outer blood–retinal barrier. The resulting permeability increase seems to induce photoreceptor apoptosis due to light exposure. The breakdown and successive apoptosis effects can be prevented by inhibiting VEGF [42]. Results obtained in our study show an increase in VEGF expression in the retinas of rats exposed to LED-backlit tablet screen light. However, results obtained with the absorbing filter, which partially filters short wavelengths, do not show VEGF expression differences compared to control group values. However, it is important to point out that the toxic outcome of VEGF on photoreceptors is not a direct effect, but secondary to VEGF-induced RPE permeability. It seems plausible that after exposure to high levels of light, the breakdown of the epithelium integrity alters the metabolite exchange between retina and choroid, which, in turn, may further affect photoreceptors [42].

We also found after up-regulation of TNFα, SOD1, and SOD2 after exposition to LED tablet screen light. As far as we know, the regulation of the expression of TNFα by light has not been previously reported; nevertheless, this could be in agreement with the inflammatory mechanism that underlines the age-related macular degeneration induced or exacerbated by blue light [43]. Finally, the increase in SOD1 and SOD2, two enzymes intimately related to oxidative stress after light exposure, suggests a contribution to the whole retinal damage. Shang et al. observed free radical production in the retina after LED-exposition [9]. Similarly, Jaadane et al. and Nakamura et al. indicated that oxidative stress was partially involved in blue LED light-induced retinal damage [2,7].

Several studies have speculated on the damaging effects that short wavelength radiation can cause on the eye and the possible protective effect of optical filters that selectively absorb this light. In 2004, Sparrow et al. already suggested that a blue light partial absorption filter reduces approximately 80% and 78% in the death of the RPE exposed to blue (430 nm) and white light ( 5400 °K) [32]. Other studies, such as those published by Yanagi et al. in 2006 and by Hui et al. in 2009, demonstrated that the blue light partial absorption filter protects the RPE from the damage produced by short wave visible radiation, increasing cell viability by 42% and 79.5%, respectively [44,45]. On the other hand, in 2011, Zhou et al. observed that the viability of cells exposed to 430 ± 20 nm was reduced by 40% [46]. To evaluate possible artificial protection mechanisms, they interposed optical filters with different levels of a pigment that absorbs blue light and verified that the protection provided by the filter was a function of its absorbance. In 2013, Chamorro et al. published a study whose results showed that the absorption of blue light decreases apoptotic cell death by 50–89% and inhibits DNA damage by 57–81% [3].

Although all these studies have results consistent with ours, the research carried out by Chamorro et al. stands out, since, in their experiment, they used LED light sources, just like us. However, our study presents two important differences with theirs when analyzing the results. First, their light exposure system consisted of an experimental device with direct emission LEDs, while our lighting system consisted of LED-backlit screens, currently commercialized, which emit white light diffusely. It should be noted that, under normal conditions, a person can spend long hours watching screens and not specific sources of LEDs, which gives our study greater similarities with real experiences. Second, Chamorro et al. performed an in vitro experiment exposing an RPE cell culture to LED light. However, our study of animal experimentation has allowed us to expose the eyes to the LED radiation, keeping intact the ocular optical systems of protection, such as, for example, the filtering of light provided by the cornea, the lens, and the intraocular media. In addition, in our study, the retinal tissue analyzed was contained in the eye of a living animal during exposure to light, therefore, it kept intact the regeneration mechanisms of photopigments and cells that the eye presents under normal conditions of life during the exposure period. This gives our results greater consistency with the effects that blue light and selective absorbance filters for this radiation can produce on living retinal tissue.

In this study, we assessed genes involved in retinal damage in the classic model of pigmented rats. Although extrapolation of the results obtained in rats to humans is questionable, it is useful to gain knowledge on the type of detrimental effects that the new LED-backlit screens can generate. Additionally, it is important to consider that results obtained in in-vitro studies with human retinal cells are similar to those obtained in our study. Along this same line, Nagai et al. have shown that blue-light filtering intraocular lenses that absorb a percentage of short-wavelengths that reach the human retina, decrease the incidence of retinal degeneration [33].

Although further investigations are still needed, there is evidence about how LED light damage depends on the exposure time and the wavelength, and involves different pathways related to oxidative stress, inflammation, ECM degradation, regulation of apoptotic genes, and growth factors. As LED-backlit screens are watched at a short distance, we stress the importance of controlling excessive exposure to LED light currently imposed by the widespread use of digital devices such as computers, laptops, tablets, smartphones, etc. The use of short-wavelength selective filters on screens may contribute to reducing potentially irreversible damage to the human retina, by partially cutting off the excessive and most energetic blue-light emissions.

## Figures and Tables

**Table 1:** Genes sequences analyzed in this study.

	Accession Number	Forward	Reverse
**Life/Death**			
Bcl-2	NM_016993	5′-ATCTTCTCCTTCCAGCCTGA3′	5′-CTGGACATCTCTGCAAAGTC3′
Bcl-xl	BC094213	5′-AAGAAACTGAACCAGAAAGG3′	5′-TAGATCACTGAATGCTCTCC3′
Bax	NM_017059	5′-CTACAGGGTTTCATCCAGGA3′	5′-ATCCACATCAGCAATCATCC3′
Bak	AF259504	5′-CTATTTAAGAGCGGCATCAG3′	5′-ATTACCACTACACTCAGGAT3′
Bcl-xs	AF279286	5′-CGGAGAGCATTCAGTGAT 3′	5′-CCAGCAGAACTACACCAG 3′
Caspase 3	NM_012922	5′-GTCATGGAGATGAAGGAGTA3′	5′-AGAGTAAGCATACAGGAAGT3′
Caspase 9	NM_031632	5′-GTCTGTACTCCAGGGAAGAT3′	5′-TTAGCAGTCAGGTCGTTCTT3′
**ECM degradation**			
MMP2	NM_031054	5′-GCAATACCTGAACACTTTCT3′	5′-ATCTGATTCTTGTCCCACTT3′
MMP9	NM_031055	5′-GACTACGACACAGACAGAAA3′	5′-GAGTAGGACAGAAGCCATAC3′
ADAMTS-12	NM_001106420	5′-CTGCCAGAATACCACATAGT3′	5′-TATCTCCTCTCCACGACATA3′
ADAMTS-14	NM_001107636	5′-GCTACCTCCTATCCTACAAT3′	5′-CTTGGTCTTGCAGAAGTATG3′
TIMP1	U06179	5′-CCACCTTATACCAGCGTTAT3′	5′-CTGGGACTTGTGGACATATC3′
TIMP22	NM_021989	5′-AGATGTTCAAAGGACCTGAC3′	5′-CTTCTTCTGGGTGATGCTAA3′
**Growth factors**			
BDNF	NM_001270630	5′-GTGACAACAATGTGACTCCA3′	5′-CATTCACGCTCTCCAGAGTC3′
TrkB	AY265419	5′-CCAGAGAACATCACCGAAAT3′	5′-ATCAGGTCAGACAAGTCAAG3′
VEG	NM_001110333	5′-GTATATCTTCAAGCCGTCCT3′	5′-CATTCACATCTGCTATGCTG3′
VEGFR2	NM_013062	5′-GGCAAATACAACCCTTCAGA3′	5′-CCGATAGAAGCACTTGTAGG3′
TNFα	NM_012675	5′-GCTCTTCTGTCTACTGAACT3′	5′-CTTTGAGATCCATGCCATTG3′
**Oxidative stress**			
SOD1	NM_017050	5′-CTTTGAGATCCATGCCATT3′	5′-ACACGATCTTCAATGGACAC3′
SOD2	NM_017051	5′-GAGAACCCAAAGGAGAGTTG3′	5′-CTGAAGATAGTAAGCGTGCT3′
**β-actin**	NM_031144	5′-ATCGTGCGTGACATTAAAGA3′	5′-GATGCCACAGGATTCCATAC3′

**Table 2:** Antibodies used in the study.

Antigen (Clone)	Origin	Dilution	Supplier
**Life/Death**			
Bcl-2 **Cat #** PA5-11379	Rabbit	1:200	ThermoFisher ^1^
Bcl-xl **Cat #** PA5-17805	Rabbit	1:100	ThermoFisher ^1^
Caspase 3 **Cat #** PA5-77887	Rabbit	1:400	ThermoFisher ^1^
Caspase 9 ab25758	Rabbit	1:200	Abcam ^2^
**ECM degradation**			
MMP2 (clone MMP2/2C1) LS-C2814-100	Mouse	1:100	LifeSpan Biosciences ^3^
MMP9 ab38898	Rabbit	1:200	Abcam ^4^
**Growth factors/Cytokines**			
TrkB catalog # sc-12	Rabbit	1:100	Santa Cruz Biotechnol ^5^
VEGFR2 PA5-16487	Rabbit	1:100	Invitrogen ^6^
**Oxidative stress**			
SOD1 Ab13498	Rabbit	1:200	Abcam ^4^
SOD2 ab13533	Rabbit	1:100	Abcam ^4^

^1^ Watham, MA, USA; ^2^ Abcam: Cambridge, UK; ^3^ Seattle, WA, USA; ^4^ Santa Cruz, CA, USA; ^5^ Dallas, TX, USA; ^6^ Waltman, MA, USA.

**Table 3:** Changes in the gene expression in the two established experimental groups in relation to the controls.

Gene Expression	Group 1 Average vs. Control	Group 2 Average vs. Control
**Life/Death**		
Bcl-2	−12.6 ± 2.71	−2.7 ± 0.3
Bcl-xl	−3.4 ± 0.4	0.4 ± 0.01
Bax	5.2 ± 0.86	2.2 ± 0.2
Bak	3.6 ± 0.63	0.1 ± 0.0
Bcl-xs	1623 ± 2.22	2.3 ± 0.61
Caspase 3	6.1 ± 1.03	0.0 ± 0.0
Caspase 9	11.3 ± 1.37	3.9 ± 0.83
**ECM degradation**		
MMP2	13.1 ± 2.65	2 ± 0.33
MMP9	16.3 ± 3.21	3.3. ± 0.39
ADAMTS-12	0.0 ± 0.0	0.0 ± 0.0
ADAMTS-14	4.6 ± 0.99	1.1 ± 0.0
TIMP1	−6.4 ± 1.91	−2.1 ± 0.3
TIMP22	−4.8 ± 1.27	−1.9 ± 0.52
**Growth factors**		
BDNF	0.0 ± 0.0	0.0 ± 0.0
TrkB	7.1 ± 1.42	3.1 ± 0.66
VEG	2.3 ± 0.25	0.0 ± 0.0
VEGFR2	11.5 ± 2.84	4.4 ± 0.98
TNFα	14.4 ± 2.72	1.7 ± 0.23
**Oxidative stress**		
SOD1	24.6 ± 6.33	5.3 ± 1.75
SOD2	19.3 ± 3.77	6.1 ± 1.42

**Table 4:** Variations in the intensity of immunostaining in the control and the two experimental groups.

Retinal Layer	Protein	Control	LED	LED + F
**Photoreceptors**	Blc2	+/++	−/+	+
	BclX	−	−	−
	Caspase 3	+	+/++	+
	MMP9	−	−/+	−
	TrkB	+/++	+++	++
	VEGFR2	−	−	−
	SOD1	−/+	++	+
	SOD2	+	+++	+/++
**ONL**	Blc2	++	−	−/+
	BclX	+/++	+/++	+/++
	Caspase 3	−/+	+/++	−/+
	MMP9	+/++	+/++	+/++
	TrkB	+/++	+/++	+/++
	VEGFR2	+	++	++
	SOD1	+	+	+
	SOD2	+	++/+++	++
**INL**	Blc2	++	−	−
	BclX	+/++	+/++	+/++
	Caspase 3	−	+/++	−
	MMP9	++	++/+++	++/+++
	TrkB	+/++	+++	++
	VEGFR2	+	++	+/++
	SOD1	−/+	−/+	−/+
	SOD2			
**Ganglionic cells**	Blc2	+++	+	++
	BclX	++	++	++
	Caspase 3	+/++	++/+++	−/+
	MMP9	++	+++	+++
	TrkB	++/+++	+++	+++
	VEGFR2	++	++	++
	SOD1	++/+++	+	++/+++
	SOD2	+/+	+++	+++

## Data Availability

The data that support the findings of this study are available from corresponding author upon reasonable request.
